# The effect of electronic medical record system use on communication between pharmacists and prescribers

**DOI:** 10.1186/s12875-015-0378-7

**Published:** 2015-10-28

**Authors:** Alexander Singer, Roberto Duarte Fernandez

**Affiliations:** Department of Family Medicine, University of Manitoba, Winnipeg, Manitoba Canada

**Keywords:** Electronic health record, Electronic medical record, Electronic medical records, Electronic health records, Prescribing, Primary care, Medication safety

## Abstract

**Background:**

The Electronic Medical Record (EMR) is becoming increasingly common in health care settings. Research shows that EMRs have the potential to reduce instances of medication errors and improve communication between pharmacists and prescribers; however, more research is required to demonstrate whether this is true. This study aims to determine the effect of a newly implemented EMR system on communication between pharmacists and primary care clinicians.

**Methods:**

A retrospective chart analysis of primary care EMR data comparing faxed pharmacy communications captured before and after the implementation of an EMR system at an academic family medicine clinic. Communication requests were classified into the following various categories: refill accepted, refill denied, clarification, incorrect dose, interaction, drug insurance/coverage application, new prescription request, supplies request, continued care information, duplicate fax substitution, opioid early release request, confirmation by phone call, and other.

**Results:**

The number and percentage of clarification requests, interaction notifications, and incorrect dose notifications were lower after the implementation of the EMR system. The number and percentage of refills accepted and new prescription requests increased after the implementation of the EMR system.

**Conclusion:**

The implementation of an EMR in an academic family medicine clinic had a significant effect on the volume of communication between pharmacists and prescribers. The amount of clarification requests and incorrect dosing communications decreased after EMR implementation. This suggests that EMRs improve prescribing safety. The increased amount of refills accepted and new prescription requests post EMR implementation suggests that the EMR is capable of changing prescription patterns.

## Background

Medication errors are an unfortunate, yet frequent part of medical care. Franklin et al. [1] define medication errors as “any error in prescribing, dispensing or administration of medication.” These are common causes of preventable drug-related emergency department visits, hospitalizations and deaths. In some cases, these errors may result in adverse drug events, which refer to any harm induced by medication administered during medical treatment or diagnostic procedures [1]. According to Kohn et al. [2], adverse events cost the United States between $37.6 billion and $50 billion, while preventable adverse events cost them between $17 billion and $29 billion. These values include costs sustained by lost income, lost household production, disability, and health care costs. In 1993, medication errors were responsible for an estimated 7,000 deaths [2]. In a study in Germany, Rottenkolber et al. [3] found that adverse drug events cost the country €1.058 billion annually. In Canada, preventable drug-related hospitalizations cost the healthcare system an estimated $2.6 billion per year [4].

There are different types of medication errors, one type being prescribing errors [1]. Many medication errors may be related to pharmacists misinterpreting the exact details on a prescription. Presumably this was a greater issue with hand written prescriptions and digital health records should improve this type of error. In a study carried out in the UK, researchers found that 36% of paper records were at least somewhat illegible, while legibility of the EMR was not an issue [5]. Prescribing errors made up 70% of medication errors in two studies carried out by the American Academy of Family Physicians and National Research Network in primary care clinics; according to Kuo et al. [6], electronic tools could have prevented at least half of these errors. Based on these studies, it is clear that prescribing involves a significant risk for patients. Fortunately, communication between prescribing clinicians and pharmacists is evolving and changing with the expanded and consistent use of electronic medical records (EMR) in primary care.

Studies examining the effect of EMRs on prescribing have shown an impact on prescribing practices and potential errors. Based on the findings of Lau et al. [7], EMR use has a 51% chance of improving office practice, a 19% chance of having negative consequences, and a 30% chance of not having any effect. In a study in Pakistan, electronic prescribing had a profound impact on prescribing errors and decreased their frequency [8]. According to Tamblyn et al. [9], the use of computerized decision-making support resulted in fewer instances of inappropriate prescriptions and higher rates of discontinuation of drugs causing harmful interactions. While it is clear that EMRs have the potential to improve quality of care by reducing the likelihood of errors, further exploration is needed to determine if this potential is being met.

The purpose of this study is to determine the effect of a newly implemented EMR on communication between pharmacists and primary care clinicians. There is a paucity of data on the effect of EMRs on communication between prescribers and pharmacists in primary care populations. Our aim was to determine how the EMR affects the number and type of faxed communication requests received from pharmacists. During the study period, there were no formal changes to the typical practices and normative behaviours regarding how clinicians decided on whether to issue a prescription without seeing a patient. As in other clinical environments however, it is a clinician’s decision based on the information available whether to issue a renewed prescription without formally seeing a patient in the clinic. We hypothesized that after several years of using an EMR, communication patterns and types of communication will have changed. Specifically, we were most interested in whether the introduction of EMRs would reduce the number of clarification faxes. By better understanding this process, we may also be better able to expect the impact of further modernization on prescribing in the primary care environment.

## Methods

We conducted a retrospective chart analysis comparing faxed pharmacy communications captured before and after the implementation of an EMR in December 2011 at a family medicine academic teaching unit in Winnipeg. We analyzed faxed pharmacy communications spanning from September to November 2011 (the period immediately prior to the implementation of the EMR) and we analyzed communications from one of the provincially approved EMRs (QHR Accuro) from September to November 2014 (39 months after EMR implementation). Requests were classified into various categories including: refill accepted, refill denied, clarification, incorrect dose, interaction, drug insurance/coverage application, new prescription request, supplies request, continued care information, duplicate fax, substitution, opioid early release request, confirmation by phone call, and other. Clarification requests were defined as requests that could not be interpreted by pharmacists without intervention because of illegibility or other communication issues. The analysis was conducted at the Family Medical Centre (FMC) in the Department of Family Medicine at the University of Manitoba. FMC is an academic family medicine teaching unit that has prescribers including primary care clinicians (Family Physicians and Nurse Practitioner) along with resident learners and a clinician pharmacist, as well as other members of a multi-disciplinary health team including a nurse, dietician and social worker. Ethical approval for this study was obtained from the Winnipeg Regional Health Authority and the University of Manitoba Research Ethics Board. Individual participant consent was not obtained in accordance with Canada’s Tri-Council Policy Statement: Ethical Conduct for Research Involving Humans and the University of Manitoba Research Ethics board policy regarding retrospective chart reviews.

## Results

Our findings are summarized in Table 1, consisting of our raw data. Our statistical findings are summarized in Tables 2 and 3.

**Table 1:** Different categories of communication between pharmacists and prescribers, and their respective amounts before EMR implementation and three years after EMR implementation

Request category	Pre-EMR live date (555 requests)	Post-EMR live date (857 requests)
Refill Accepted	259 (46.7%)	497 (58.0%)
Refill Denied	21 (3.8%)	32 (3.7%)
Clarification	64 (11.5%)	50 (5.8%)
Incorrect Dose	29 (5.2%)	13 (1.5%)
Interaction	3 (0.5%)	1 (0.1%)
Exception Drug Status (EDS) Request	25 (4.5%)	19 (2.2%)
New Rx Request	74 (13.3%)	160 (18.7%)
Supplies Request	13 (2.3%)	21 (2.5%)
Continued Care Information	2 (0.4%)	6 (0.7%)
Duplicate Fax	3 (0.5%)	11 (1.3%)
Substitution	16 (2.9%)	16 (1.9%)
Fill Over Phone	16 (2.9%)	0 (0.0%)
Opioid Early Release Request	5 (0.9%)	6 (0.7%)
Other	25 (4.5%)	25 (2.9%)

**Table 2:** Results of a Pearson’s chi-square test conducted for all categories, where DF stands for degrees of freedom and the sample size was 1,412

Statistic	DF	Value	*P* value
Chi-Square	13	82.1595	<0.0001

**Table 3:** Results of a Pearson’s chi-square test conducted for clarification requests compared to all other requests, where DF stands for degrees of freedom and the sample size was 1,412

Statistic	DF	Value	*P* value
Chi-Square	1	14.7318	0.0001

Table 1 presents the number of different communication requests between pharmacists and prescribers, both before electronic medical records (EMRs) were implemented and three years after EMRs were implemented. In both time periods the most frequent communications were regarding accepted refills, while the least frequent communications concerned drug interactions. In the refill accepted, refill denied, new prescription, supplies request, continued care information, duplicate fax, and opioid early release request categories there were more communications after EMR implementation than before EMR implementation. Table 1 also shows that the number of clarification and incorrect dose communications was lower after EMR implementation Tables 4 and 5.

**Table 4:** Table listing request categories and their definitions

Request categories	Definitions
Refill Accepted	Approved refill request.
Refill Denied	Rejected refill request.
Clarification	Requests requiring physician intervention to interpret. Ex: prescription illegible.
Incorrect Dose	Dosage prescribed by prescriber did not match what pharmacists had on record.
Exception Drug Status Request	Application for drug insurance or coverage.
New Rx Request	Request for prescription not on file for that patient.
Supplies Request	Request for non-drug equipment. Ex: diabetic equipment such as touch strips.
Continued Care Information	Requests for refills or new prescriptions relating specifically to individuals in continuing care communities.
Duplicate Fax	Copies of the same fax passed through the system more than once.
Substitution	Request for an alternate medication to replace a current one.
Fill Over Phone	Request that was completed in a phone interaction between pharmacist and prescriber.
Opioid Early Release Request	Application for release of opioid drugs to patient ahead of their intended time. Ex: patient is going away and would like to fill their hydrocodone prescription early.
Other	See Table 5.

**Table 5:** Description of categories counted as “other,” EMR era in which they appeared, and the number of each

Other categories	Pre or Post EMR	Total number of each
Discontinued prescription	Pre	3
Update clinical records		1
Compliance packaging		2
Request unclear		1
Early refill for non-opioid		7
Blisterpack request		3
Patient allergy		1
Clinical error		1
Missing prescriber signature		4
Patient no longer seen		1
Error by pharmacy		1
Follow up request from doctor	Post	1
Patient requests dose change		5
Notification of pharmacist authorized prescription		3
Home care program		9
Missing signature		3
Flu shot		1
Individual not a patient		1
Limited use request form		1
Physician initiated communication		1

Pearson’s chi square test was used to compare the use of an EMR and different request categories. This revealed that the use of an EMR did have an impact on the difference in the number of requests seen in both timeframes, *p* < 0.0001. In a separate Pearson’s chi square test, the use of an EMR was shown to affect the number of clarification requests, specifically, in each timeframe, *p* = 0.0001. In both tests, our p-values indicate statistical significance. PROC GLIMMIX of SAS version 9.3 (SAS Institute, Cary NC) was used for our analysis.

## Discussion

Our results demonstrate a statistically significant change in the types of faxed communication between pharmacists and primary care providers after the implementation of an EMR. The most clinically significant change is a dramatic reduction in the number of incorrect dose and clarification requests, with a slight decrease in the number of interaction requests. It is also interesting to note that the number of refill requests and duplicate faxes increased after EMR implementation.

The reduction in clarifications and incorrect dose notifications from pharmacists supports previous work demonstrating that EMR prescriptions are consistently legible. Ammenwerth et al. [10] demonstrated that compared to handwritten prescriptions, use of EMRs led to a higher relative risk reduction in studies that compared different EMR systems. This is not surprising since EMRs eliminate the possibility of legibility errors, but from a medication safety point of view this is an important benefit. Clarification requests serve as a corollary for “near misses” in prescribing errors, as those are items that a pharmacist takes the time to review and require intervention from the pharmacist [11]. Presumably, given the large number of prescribing errors that are known to be made in general, many relate to incorrect doses or incorrect medications being dispensed, leading to patients being harmed [6].

Interactions are another area of interest. The EMR used in the study site does have a decision support tool that scans for drug interactions as do pharmacy systems in Manitoba. The number of interactions noted by pharmacists dropped, but a very small number were reported as faxed communications in both pre and post EMR implementation. This may relate to other forms of communication being used to discuss interactions, such as telephone calls. Alternatively, it may mean that the decision support systems meant to pick up interactions are not being monitored carefully and therefore, providers are not discussing the findings frequently. The dramatic increase in the total number of refill requests and requests for new prescriptions was an interesting and surprising finding. We expected the EMR to be more efficient than a paper record in terms of renewing medications at the time of a visit and determining when medications were due. We attempted to allow for significant maturity of use post EMR implementation so that primary care provider familiarity with the EMR was not as great an issue. Unpublished quality improvement work undertaken at the study site has shown that billings and appointment availability went up post-EMR, despite the clinic being mostly closed to new patients, with the exception of prenatal and pediatric patients. Practice sizes remained very stable at the study site. In addition, the total number of patient visits per month remained stable over the two time periods. Thus, there must be other potential explanations beyond lack of patient access.

One such explanation is the ease with which prescribers can fill refill requests in the new electronic system. Rather than have a patient book an appointment to fill an ongoing prescription, prescribers may be opting to examine their patients’ records and fill the refill request digitally when appropriate to do so. Our study was also not designed to consider a change in pharmacist practice during the study period either. It is also possible that during the study period there was a change in pharmacists’ communication patterns making them more likely to send faxed communications, but our study was not designed to explain such an association.

Another possible explanation may be a capture bias. The process by which fax renewal requests were recorded in the paper environment was a workflow designed by the administrative staff to keep back-up copies of prescription faxes for 1 month prior to being shredded. This was a manual process due to frequent errors requiring multiple faxes to be sent before filing the final copy in the paper chart. It is possible that some faxes did not get placed in the “back-up” binder. Conversely, when reviewing fax renewal requests in the EMR, all pharmacy faxes were stored in a structured way. Therefore, it may simply be an artifact of better capture that led to the increase, which also suggests underreporting of the other types of communication in the paper environment, strengthening our finding that the EMR improved patient safety.

The other possible explanation is the lack of a truly integrated and interoperable prescribing system. While this remains the norm in Canada for the most part, true “ePrescribing” does not exist in the study clinic. By capturing what is essentially a paper process on a computer, it is possible that the increased volume of fax renewal and new requests occurred because users did not utilize the prescribing functionality efficiently in the EMR, therefore missing the opportunity to prescribe medications at a visit. We did not have enough data to establish this as a possible cause; however, further qualitative work or advances in technology could help better explain this phenomenon.

It is also possible that some communication between pharmacists and prescribers was missed in both pre and post EMR workflows. If a pharmacist had called the clinic and spoken directly with a prescriber, this interaction would not have been captured as a distinct faxed communication document. While chart access was far less efficient in the paper environment, we have no data that suggests more or less phone calls were made in either situation. Our data may reflect a change in pharmacist practice over the two time periods related to the dramatic rise in EMR use in Manitoba during that time or other changes in primary care. There may be other factors that changed the likelihood of pharmacists to send faxed communications to prescribers but analysis of those was out of scope of this study.

Our findings are important because they demonstrate a significant change in prescribing patterns and improvement in safety with the implementation of an EMR. The implication may be that prescribing using written paper is less safe than prescribing using a computer. Various studies have noticed substantial reductions in the rates of prescribing errors after implementing computerized provider order entry (CPOE) systems [12–14]. In one study, however, certain errors increased in frequency after the CPOE system was introduced [12–14]. Interestingly, among the increased errors observed were an increase in duplicate prescriptions, which was something also observed in our study.

In our study, we observed that the number of duplicate faxes increased, despite the number of incorrect dose, interaction notification, and clarification requests decreasing. This finding has also been observed in other studies. As previously mentioned, Colpaert et al. [12–14] also found an increase in the number of duplicate prescriptions, as well as other errors, despite the decrease in the overall prescribing error rate when using a CPOE system. Senholzi and Gottlieb [15] also found that the number of duplications increased, while the number of illegible handwriting concerns decreased after implementing a CPOE system. They thought a possible explanation for this was prescribers overlooking the duplicate-therapy warning as it was not readily visible on the screen before corrections were made to the system. Another possible explanation for this increased presence of duplicate faxes comes from over-completeness associated with CPOE systems; as ease of use and the number of standard phrases increases, users are more likely to copy and paste patient data into different fields, resulting in duplication errors [16]. Interestingly, in a different study, George and Austin-Bishop [17] found that most of the errors made by physicians at the clinic of study were duplicate orders.

### Limitations

Our study did have limitations. First, our study focuses on the prescriber perspective, since we investigated the EMR in a clinical setting; as such, we lacked a direct interface between the prescribers and pharmacists and depended on fax communications to gain the pharmacists’ perspective. Second, much of the literature in this area does not use the same methods and definitions. Additionally, studies evaluating CPOE systems may not relate exactly to our setting as we lacked a direct interface between the prescriber and pharmacist in our study. As such, comparisons between studies should be made carefully [1].

There were some limitations based on our sample and retrospective design which did not allow for comparison at multiple time points to determine if the pattern observed related precisely to EMR implementation. The results are also limited to a single practice and there is no control so it is possible that this experience is not universal although other literature in this area suggests otherwise.

Our research implies that more robust prescribing systems that better integrate prescribers and pharmacists may lead to further improvements. While other industries have moved to advanced fully digitized point of contact systems that integrate consumer and warehouse systems to manage inventory, prescribing uses pre-20^th^ century technology such as faxes and printed pages signed with a pen. Our data supports investment in more advanced fully digitized prescribing with web-based database links between pharmacists and prescribers. If communication can be made more legible and safer by simply upgrading to a printed page or fax, presumably complete interoperability would offer further advantages.

## Conclusions

We found that the implementation of an EMR in an academic family medicine clinic significantly changed the volume of communication between pharmacists and prescribers in significant ways. Crucially, the amount of clarifications and incorrect dosing communications were reduced suggesting that EMRs improve prescribing safety. Conversely, the number of refill requests and requests for new prescriptions rose post EMR implementation. This may represent improved capture or propensity of the EMR to change prescription patterns at patient visits. These findings are important as they establish that EMRs may have a beneficial impact on patient safety and efficiency. By reducing the amount of time pharmacists spend clarifying prescriptions, workflow can be greatly improved. This is greatly beneficial in a community pharmacy setting where it is common to find only one pharmacist on duty at certain times in the day. Further technological advances in how prescriptions are created, managed, and how pharmacists and prescribers communicate should take improving efficiency and reducing error into account as important benefits. These data suggest an overall positive change post EMR implementation with further improvements in prescribing technology needed to fully realize the benefits digitization in primary care.

## Abreviations

AS: Alexander Singer
CPOE: Computerized Patient Order Entry
EMR: Electronic Medical Record
EMRs: Electronic Medical Records
FMC: Family Medical Centre
MaPCReN: Manitoba Primary Care Research Network
PROC GLIMMIX: “Procedure” for fitting “Generalized Linear Mixed Models”
RDF: Roberto Duarte Fernandez
SAS: Statistical Analysis System
UK: United Kindom
